# *Pseudomonas aeruginosa* PAO1 Preferentially Grows as Aggregates in Liquid Batch Cultures and Disperses upon Starvation

**DOI:** 10.1371/journal.pone.0005513

**Published:** 2009-05-13

**Authors:** David Schleheck, Nicolas Barraud, Janosch Klebensberger, Jeremy S. Webb, Diane McDougald, Scott A. Rice, Staffan Kjelleberg

**Affiliations:** 1 Centre for Marine Bio-Innovation, School of Biotechnology and Biomolecular Sciences, University of New South Wales, Sydney, New South Wales, Australia; 2 Centre for Water and Waste Technology, School of Civil Engineering, University of New South Wales, Sydney, New South Wales, Australia; Massachusetts General Hospital, United States of America

## Abstract

In both natural and artificial environments, bacteria predominantly grow in biofilms, and bacteria often disperse from biofilms as freely suspended single-cells. In the present study, the formation and dispersal of planktonic cellular aggregates, or ‘suspended biofilms’, by *Pseudomonas aeruginosa* in liquid batch cultures were closely examined, and compared to biofilm formation on a matrix of polyester (PE) fibers as solid surface in batch cultures. Plankton samples were analyzed by laser-diffraction particle-size scanning (LDA) and microscopy of aggregates. Interestingly, LDA indicated that up to 90% of the total planktonic biomass consisted of cellular aggregates in the size range of 10–400 µm in diameter during the growth phase, as opposed to individual cells. In cultures with PE surfaces, *P. aeruginosa* preferred to grow in biofilms, as opposed to planktonicly. However, upon carbon, nitrogen or oxygen limitation, the planktonic aggregates and PE-attached biofilms dispersed into single cells, resulting in an increase in optical density (OD) independent of cellular growth. During growth, planktonic aggregates and PE-attached biofilms contained densely packed viable cells and extracellular DNA (eDNA), and starvation resulted in a loss of viable cells, and an increase in dead cells and eDNA. Furthermore, a release of metabolites and infective bacteriophage into the culture supernatant, and a marked decrease in intracellular concentration of the second messenger cyclic di-GMP, was observed in dispersing cultures. Thus, what traditionally has been described as planktonic, individual cell cultures of *P. aeruginosa*, are in fact suspended biofilms, and such aggregates have behaviors and responses (e.g. dispersal) similar to surface associated biofilms. In addition, we suggest that this planktonic biofilm model system can provide the basis for a detailed analysis of the synchronized biofilm life cycle of *P. aeruginosa*.

## Introduction

Biofilms are defined as matrix-enclosed bacterial populations that are adherent to each other and/or to surfaces or interfaces [1], and have been investigated in great detail in recent years due to their significance in medical, industrial, and environmental settings. The environmental bacterium and opportunistic human pathogen *Pseudomonas aeruginosa* PAO1 is one of the best studied model organisms for bacterial biofilm formation. In the multi-cellular, surface-associated life style of this bacterium, the organism attaches to a solid surface and grows embedded in a self-generated extracellular polymeric matrix (EPM). The process of biofilm formation consists of several distinct phases, i) initial adherence of motile planktonic cells onto a surface, (ii) irreversible attachment onto the surface and (iii) unstructured biofilm growth through cell division and EPM synthesis, which at a later stage (iv) matures into a more complex three-dimensional architecture of microcolonies and void spaces [2], [3]. The study of the dispersal of *P. aeruginosa* cells from biofilms, i.e. the switch back to the planktonic lifestyle, has identified carbon starvation and oxygen limitation, as well as sudden up-shifts in carbon-substrate concentration, as major triggers for massive biofilm dispersal events [4]–[9], and a role of nitric oxide [10] and of unsaturated fatty acids [11] in inducing a biofilm dispersal response in *P. aeruginosa*. Furthermore, cell death within biofilm microcolonies has been shown to mediate biofilm dispersal, through dissolution of the biofilm matrix and release of surviving cells from the microcolonies, and filamentous bacteriophage has been suggested to regulate biofilm killing [12], [13]. For a range of bacteria including *Pseudomonas* spp., the intracellular secondary messenger cyclic di-GMP has been implicated in the regulation of transitions between the sessile and planktonic lifestyle [14]–[18].

The advantages of the surface attached multi-cellular lifestyle of bacteria in biofilms are evident in aqueous natural, industrial, and medical environments, where cells in biofilms persist (e.g. resilience against cleaning or grazing), and are increasingly tolerant to various environmental or artificial stresses and antibiotic treatments, when compared to bacteria living as dispersed planktonic single cells.

The formation of EPM-embedded cellular aggregates that are suspended in the aqueous phase, i.e. the growth of bacteria in freely suspended biofilms in flocs, is also a successful strategy used by bacteria to inhabit the pelagic zone of oceans or bodies of fresh water (marine or lake snow) [19], [20], is industrially vital, e.g. in sewage treatment plants (flocculation) [21], [22], and can be a response of bacteria to stressful environmental conditions [23], [24]. It is feasible that planktonic cellular aggregation is mediated by the same mechanisms as those involved in the formation of surface-attached biofilms, and many of the physiological traits exhibited by biofilms have been observed in planktonic cultures [25]. Therefore, the study of planktonic aggregates might be a valid model system to address contemporary questions on mechanisms and factors which mediate and modulate the formation, maturation, or dispersal of surface-attached biofilms. However, for the model biofilm bacterium *P. aeruginosa* PAO1, the extent to which planktonic cultures grow in form of cellular aggregates has not been defined.

Here, we report that planktonic cultures of *P. aeruginosa*, when incubated as standard liquid cultures which are shaken or stirred, grow predominantly as macroscopic (>100 µm diameter) and microscopic aggregates (100–10 µm diameter) which disperse upon entry into stationary phase. Microscopic examination of the aggregates revealed that the bacteria are held together in a matrix of extracellular material comprised of DNA. Aggregate dissolution was associated with the dispersal of single free-swimming cells, and was found to be triggered by depletion of a limited growth substrate, such as carbon or oxygen.

## Results

### Formation of suspended cellular aggregates in planktonic batch cultures of *P. aeruginosa*

**Figure 1:**
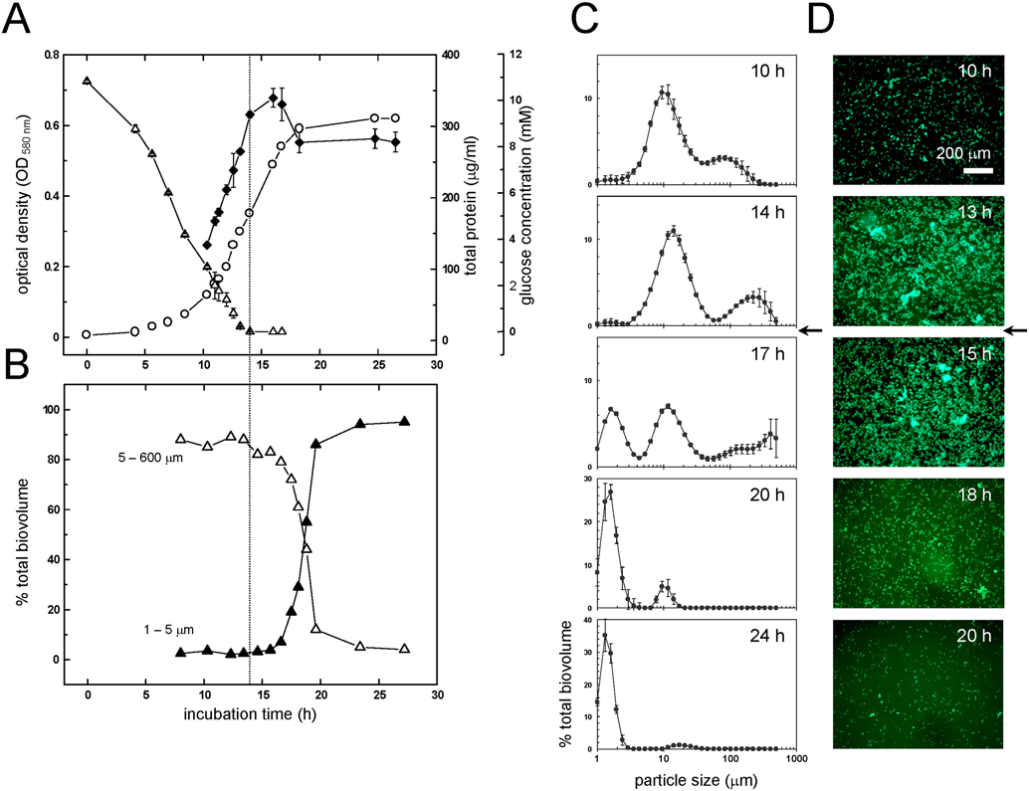
Growth and particle-size distribution of plankton in well-mixed liquid culture. The growth of a culture in the 1 L scale in a 5 L Erlenmeyer flask was monitored (panel A) for OD_580 nm_ (open circle) and total cellular protein formation (solid diamond), and disappearance of the growth-limiting substrate glucose (open triangle). The relative contribution of single cells (solid triangle) and of cellular aggregates (open triangle) to the total suspended biomass was determined by LDA (panel B). Detailed LDA particle-size scans are given of representative samples (panel C) (size range 0.5–600 µm, n = 3). The abundance of cellular aggregates during growth could also be visualized by microscopy after samples of culture fluid were allowed to sediment by gravity onto glass slides (panel D) (SYTO 9-stain, 100-fold magnification). The time point at which the culture encountered starvation due to depletion of glucose is indicated with dashed lines (panel A) and arrows (panel B and C).

**Figure 2:**
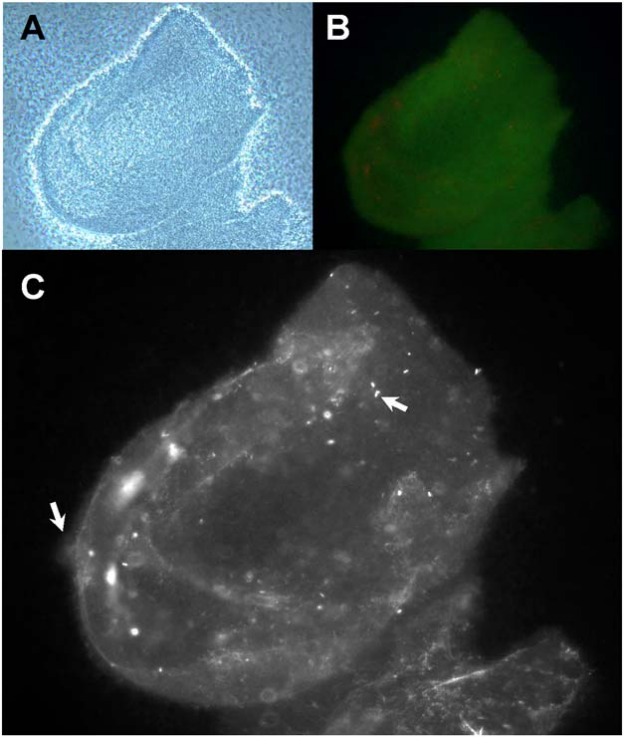
Microscopical appearance of cellular aggregates from liquid batch cultures. A GFP-labeled derivative of *P. aeruginosa* was grown in presence of a membrane-impermeable DNA stain (BOBO 3), and after 10 h incubation (cf. Figure 1), aggregates were allowed to sediment onto a microscope slide and imaged in 400-fold magnification by phase-contrast microscopy (panel A), or fluorescence microscopy (panel B, C), which showed viable cells in green and dead cells and extracellular DNA (eDNA) in red (panel B), or solely dead cells and eDNA in the red-fluorescence channel (panel C; black-and-white image). Arrows point to dead cells (right arrow) and eDNA (left arrow) in panel C.

The formation of cellular aggregates was assessed in batch cultures and observed using three different methods. *P. aeruginosa* plankton was grown in the 1 L scale in 5 L-Erlenmeyer flasks aerated by shaking at 160 rpm. First, laser diffraction analysis (LDA; see Material and Methods) was used to determine the exact size distribution of the particulate biomass in liquid culture at different phases. During the growth phase (incubation time 8–14 h, Figure 1A), approximately 90% of the total biomass consisted of particles in the size range of 5–600 µm, corresponding to cellular aggregates, whereas particles in the 1–5 µm size range, corresponding to single cells, always contributed <10% of the total biomass (Figure 1B). The particle size scans showed that two major aggregate size fractions were present after 10 to 14 h of growth, indicated as peaks with maxima at around 20 µm (size range 5–68 µm) and 200 µm (size range 68–600 µm) in diameter (Figure 1C). This represented approximately 70% and 20%, respectively, of the total biomass. However, after 19–24 h the contribution of cellular aggregates to the total biomass decreased to 10%, and single cells represented 90% of the biomass. Second, planktonic samples were allowed to sediment onto glass slides by gravity and visualized by microscopy. Prominent aggregate formation was indicated when samples were collected during the growth phase (Figure 1D), while these structures were absent in cultures that had entered stationary phase. When cultures of green-fluorescently (GFP) labeled bacteria counter-stained with a DNA-specific stain impermeable to intact bacterial membranes were observed, the aggregates contained densely packed viable cells and showed a fine-structured extracellular polymeric matrix consisting of DNA (Figure 2), and thus appeared very similar to young, unstructured surface-associated biofilms of *P. aeruginosa* (see below). Third, a sedimentation assay (centrifuge pulse) was used to remove all particles with sizes >20 µm from the plankton in samples. The centrifugation-pulsing of growth phase samples (10 h) removed >70% of the total cellular biomass, whereas after dispersal (24 h) more than 90% of the total biomass was retained in the supernatant (data not shown). Thus, it appears that under these conditions, the majority of *P. aeruginosa* suspended biomass is present as cellular aggregates and not as single, planktonic cells throughout all stages of growth, while these aggregates are mostly absent in the stationary phase.

### Dispersal of cellular aggregates in *P. aeruginosa* batch cultures upon starvation and entry into stationary phase

Glucose measurements indicated that cultures became substrate limited after 14 h incubation, at which time the total protein content of the plankton stopped increasing, and then decreased slightly (Figure 1A). Interestingly, the OD readings were found to increase further for several hours until they reached a delayed, apparent stationary phase at 17–18 h (Figure 1A). During the time between glucose depletion and stabilization of OD, LDA analysis indicated a shift of the particle size distribution from a majority of particles >10 µm to a majority of particles in the 2 µm size range, indicative of steady dispersion of cellular aggregates into single cells (Figure 1B). Further, microscopic observation of sediments collected from the culture fluid also showed that aggregates decreased both in size and number after starvation (Figure 1D). Overall these results strongly suggest that under these conditions the dominant aggregated cellular biomass in growing planktonic cultures disperses into single cells upon transition into carbon-starvation and stationary phase, and that the dispersal event is reflected by a concomitant increase of OD uncoupled from cellular growth. Thus, once starvation has been defined in these liquid cultures, by simple determination of the limiting growth substrate, the stabilization of OD after starvation can be used to monitor the dispersal phase in planktonic cultures of *P. aeruginosa* (see below).

### Dispersal of surface-attached biofilms after glucose depletion

**Figure 3:**
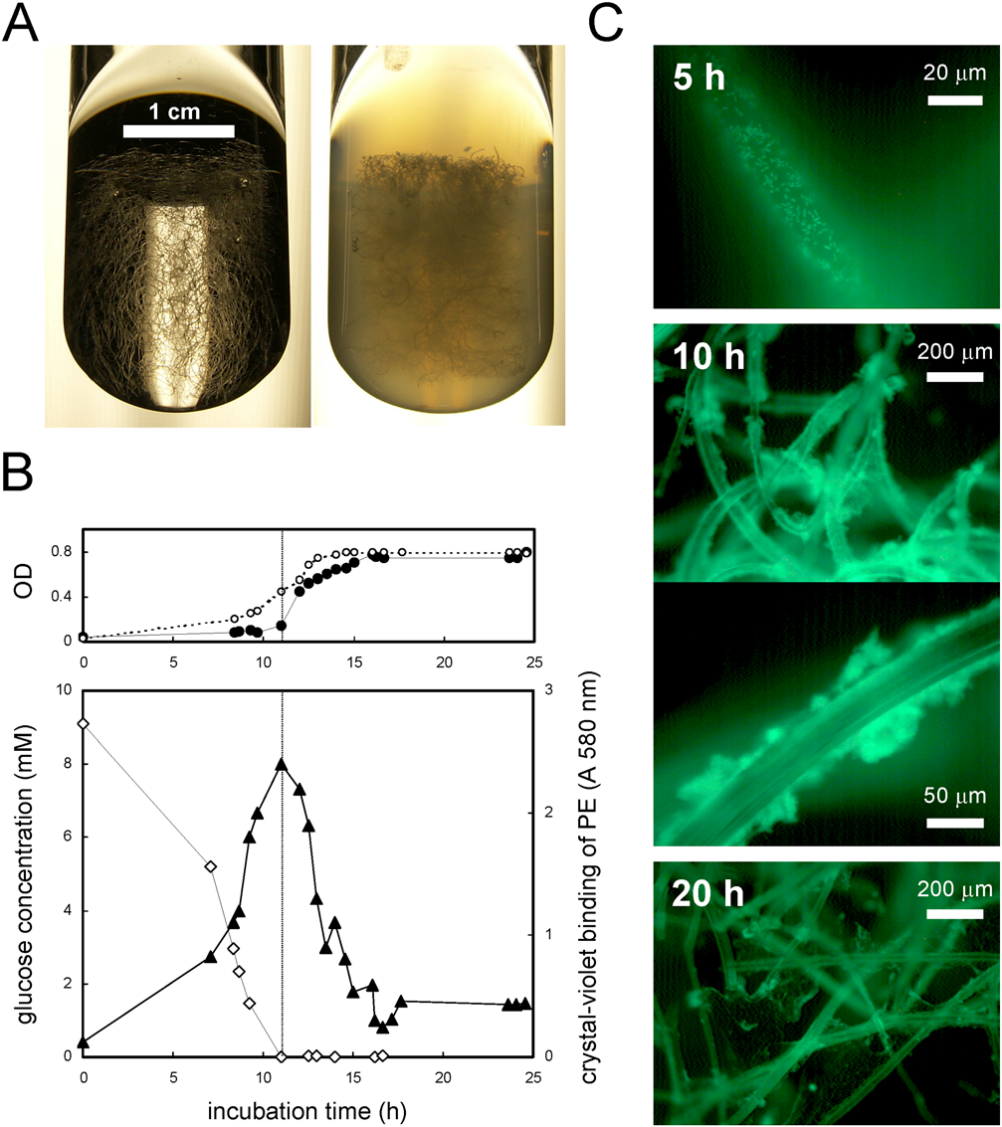
Formation and dispersal of biofilms on additional solid surface in well-shaken planktonic cultures. Biofilms were grown on polyester (PE) fleece as additional solid surface for biofilm formation, a wide-open matrix of polyester fibers which was suspended in culture fluid on an orbital shaker (140 rpm). Series of PE-cultures were inoculated in parallel, and at intervals during incubation each one tube was sacrificed to remove PE for biofilm examination, and to examine planktonic cellular biomass in the remaining culture fluid. Image of PE suspended in culture medium in glass tubes (panel A) before inoculation (left image) and in outgrown cultures (right image; after 24 h incubation). Growth and transition of the cultures into stationary phase (panel B) as followed by glucose determination (open diamond), growth and dispersion of biofilms attached to PE as quantified by crystal-violet staining (solid triangle), and the density of the freely suspended planktonic biomass in the cultures as followed by OD (inset, solid circle) when compared to the OD values obtained in control cultures without PE (inset, open circle). Microscopic appearance of biomass (SYTO 9-stain) attached to PE in the early growth phase (after 5 h), during the late-exponential growth phase (after 10 h), and in the stationary phase (after 20 h), respectively (panel C).

**Figure 4 pone-0005513-g004:**
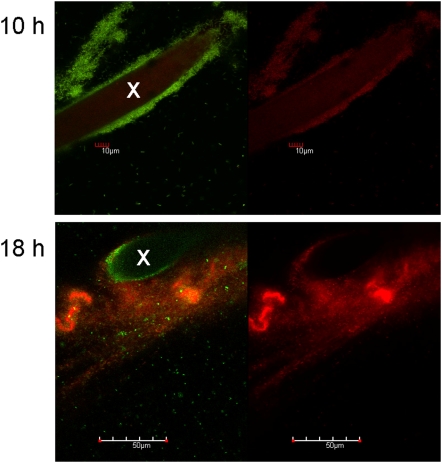
Confocal-microscopical appearance of PE-attached biofilms before and after starvation. GFP-fluorescent bacteria were grown in presence of a membrane-impermeable DNA stain (BOBO 3) and PE was imaged during the growth (10 h) and during the stationary phase (18 h). The green/red fluorescence image is given (left panel), and solely the red fluorescence channel indicative of dead cells and of extracellular DNA (right panel). Transects of PE fibers are indicated by “x”.

Starvation-induced dispersal was further studied in surface attached *P. aeruginosa* PAO1 biofilms, by using batch-culture biofilm systems as well as continuous flow-cell biofilm systems. To confirm that starvation-induced biofilm dispersal occurred for surface attached biofilms in batch cultures, pieces of polyester (PE) fleece (Figure 3A) were added as solid surface for biofilm formation [26]. It was observed that glucose was utilized and biofilms formed on the PE before any significant planktonic growth was detectable (Figure 3B), and that the total cellular-protein content of the biofilms on PE always contributed >60% relative to total cellular protein content of the whole culture before starvation (data not shown). This indicated that *P. aeruginosa* preferred to grow on the surface rather than in the planktonic phase under these conditions. After 12 h incubation, glucose was depleted and the biofilms almost completely dispersed into the planktonic phase within 4 h as indicated by the decrease in crystal violet staining of PE with a concomitant increase in OD to similar values as observed in control cultures without PE (Figure 3B). Similar results were obtained (not shown) when other carbon sources were tested (acetate, succinate), or when cultures entered stationary phase due to nitrogen limitation (3 mM NH_4_Cl). These results were supported by microscopic observation of cultures containing PE and GFP-labeled *P. aeruginosa* (Figure 3C). LDA scans on plankton samples taken during the growth phase indicated an absence of aggregates in the size class >200 µm diameter during growth (data not shown), and it may be that the >200 µm particles attached onto the PE fibers (cf. Figure 3C, 10 h). Upon starvation, the LDA bio-volume contribution of single cells increased from 20% to >90% within 30 min, reflective of fast and massive dispersal of single cells from the PE-attached biofilms (data not shown). The residual biomass on PE after starvation and dispersal had a slimy texture, which also stretched across individual PE fibers (Figure 3C, 20 h). Confocal microscopy of sections of PE using GFP-labeled bacteria counter-stained with a DNA-specific stain impermeable to intact bacterial membranes showed that biofilms after 10 h of growth were comprised of viable cells, and a faint red fluorescence indicated eDNA as constituent of the EPM (Figure 4), as observed for planktonic aggregates (cf. Figure 2). In contrast, after 18 h incubation, the biomass remaining on PE fleece exhibited increased amounts of eDNA and dead cells, and appeared to lack viable cells, indicative of dispersal events (Figure 4).

**Figure 5:**
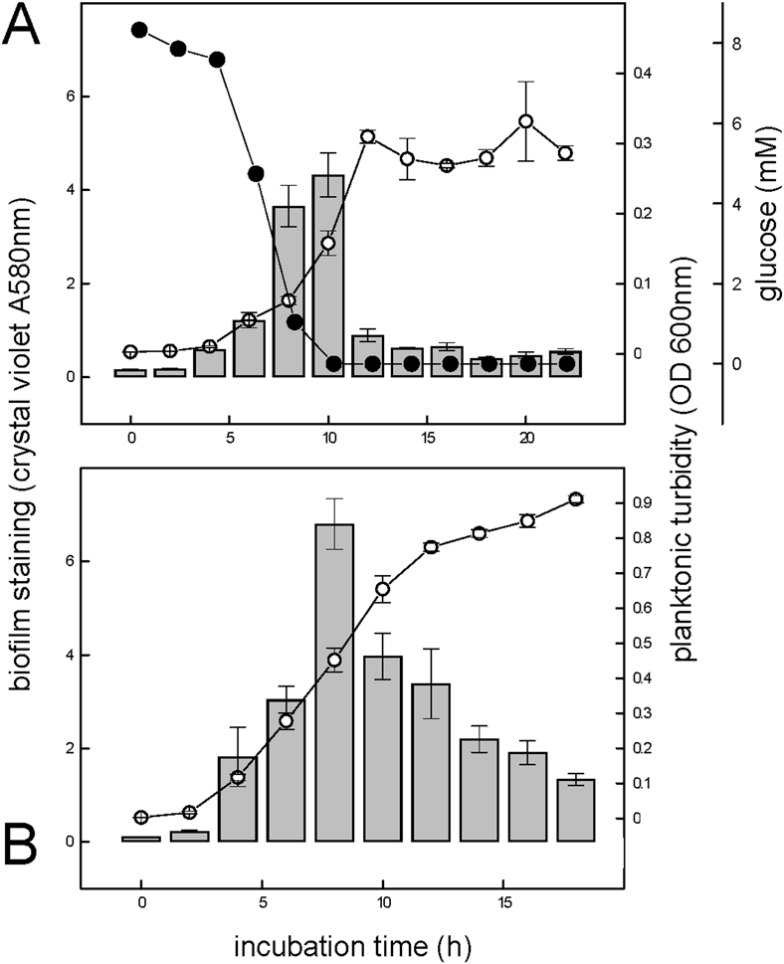
Formation and dispersal of biofilms during growth of *P. aeruginosa* in well plates. Growth of liquid cultures (n = 3) in 12-well plates on an orbital shaker (120 rpm) was monitored as glucose disappearance (solid circles), OD increase of the planktonic phase of the cultures (open circles) and as biofilm formation in the wells by crystal-violet staining assay (bars), if a glucose/mineral salts medium (M9 medium) was used (panel A). When a complex medium (LB-medium) was tested (panel B), an adequate method to analyze the concentration of bulk carbon source was not available; when samples of culture fluid were filter-sterilized and re-inoculated, no growth was observed in samples taken after 15 h incubation.

Similar effects were observed when biofilms were cultivated in 12-well microtiter plates. Surface-attached biofilm growth predominated during glucose utilization, while the biofilms dispersed into the planktonic phase after glucose starvation (after 10 h) (Figure 5A). This was also observed when cells were grown in complex medium (LB medium) (Figure 5B).

It was previously shown that artificially induced carbon starvation resulted in dispersal in continuous flow culture biofilms of *P. aeruginosa* PAO1 [5]. Therefore, biofilms were grown for two days on PE, at which time the culture medium was switched from glucose containing medium to glucose-free salts medium (data not shown). The biofilms started to detach within 30 min after glucose starvation as indicated by significant increase in OD of the planktonic phase after starvation, and microscopic visualization of PE confirmed that most of the biomass had dispersed after starvation. Interestingly, single cells and also large aggregates were observed in the biofilm effluent, which indicated that sloughing of biofilms also contributed to the biofilm dissolution event (not shown).

### Dispersal of planktonic aggregates during oxygen limitation

**Figure 6:**
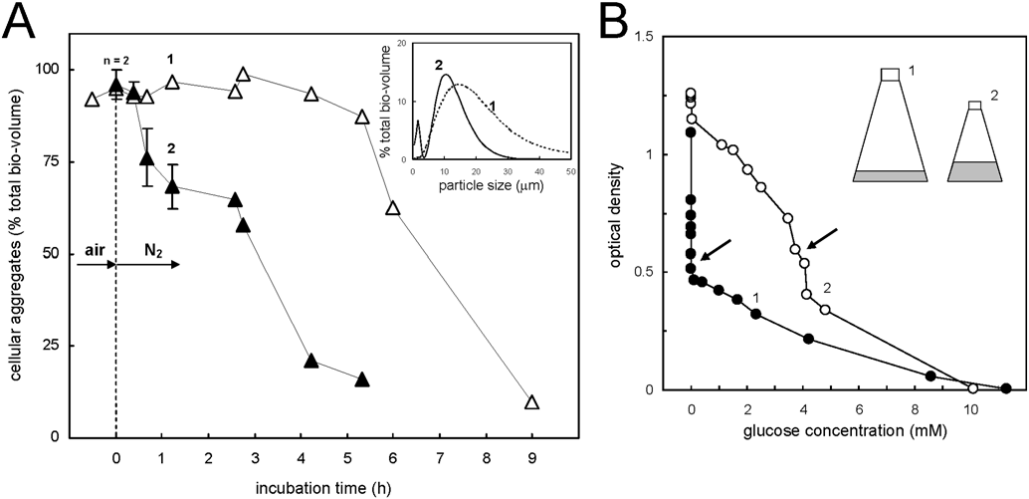
Dispersion of planktonic cellular aggregates during oxygen-limited growth. Series of cultures in glass tubes which grew in parallel were depleted in molecular oxygen (panel A) through exchange of the air phase by nitrogen gas (at 0 h), and the size distribution of the plankton was followed at intervals by LDA in comparison to a series of untreated control cultures. LDA size scans (range 0.5–50 µm) of a representative sample taken after the treatment and of an untreated control sample is given as inset, and referred to by numbering. Cellular aggregates in larger planktonic cultures (1 L scale) were observed to disperse before the depletion of glucose (panel B) when incubated in a smaller culture vessel (open circle) compared to control cultures (Figure 1) (solid circle), as illustrated here by a linearized growth plot of OD formation versus substrate utilization. Drawings of the dimensions of the liquid volume in the culture vessels are given to scale (3 L and 5 L Erlenmeyer flasks) and referred to by numbering. Arrows indicate the approximate time point at which increased foaming and yellow/green color of the culture fluid was noted (see the text).

Oxygen depletion was described as factor which induces massive biofilm dispersal events [8], [9], and we tested the effect of oxygen depletion on planktonic aggregates. When air was exchanged with nitrogen in sub-samples taken from a growing aggregated culture, and compared against untreated control sub-samples (Figure 6A), almost immediate dispersal was detectable by LDA, at a similar rate as observed during dispersal after glucose starvation (see the control in Figure 6A). This oxygen depletion effect was confirmed for biofilms in PE-containing cultures, where a fast increase of OD, and loss of crystal violet staining of PE, was observed (data not shown). The limitation of molecular oxygen during growth and dispersal could also be achieved by limiting gas exchange either through decreasing the liquid surface area, or slowing down the shaking speed. For example, when 1 L cultures were incubated in two different sized Erlenmeyer flasks, 3 L and 5 L (cf. inset of Figure 6B), the cultures in the 3 L flask showed higher OD readings relative to the amounts of glucose utilized for growth compared to the same culture volume in the 5 L flask (Figure 6B). A higher proportion of single cells in the 3 L flask, and hence dispersal of cellular aggregates earlier than in cultures incubated in larger flasks, was confirmed by LDA (data not shown).

### Compounds released into the culture fluid after starvation and dispersal of planktonic aggregates

We noted that dispersing aggregates, either in well-aerated cultures after glucose depletion (Figure 1), or in oxygen limited cultures (Figure 6), tended to adhere to plasticware, e.g. into pipette tips during sampling, and were of slimy consistency, as opposed to the dense, non-sticky aggregates observed during the growth of well-aerated cultures (not shown). The stickiness was suggested to be associated with eDNA release, based on the observations above (Figure 4D). Two additional changes were noted in the visual appearance of the culture fluid concomitant to initiation of dispersal, either after glucose depletion (Figure 1) or during oxygen-limited growth (Figure 6B), and could reliably be used to visually determine the initiation of dispersal (cf. arrows in Figure 6B). One change was the increased foaming of the culture, which was attributed to release of rhamnolipid surfactants [27] and the second was an increased yellow/green color of the culture fluid, most likely attributed to the release of pyocyanine [28]. Our visual observations were confirmed by using 0.2-µm filtrates of culture fluid to quantify the release of crude EPM- and/or cellular components into the culture fluid (total soluble DNA, sugars, and protein) and of the metabolites rhamnolipid and yellow/green pigment (see Material and Methods). The results (data not shown) indicated that, with the exception of soluble sugars, there was an increased release of all of these compounds at the time of starvation.

### Intracellular levels of secondary messenger cyclic di-GMP during growth and in the stationary phase

**Figure 7:**
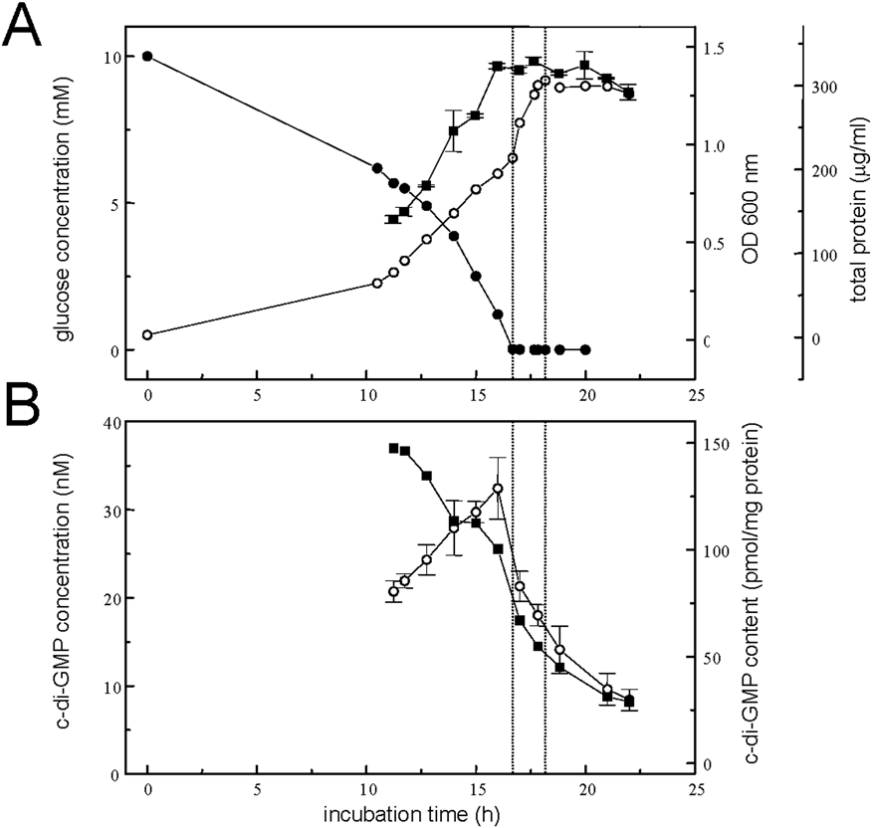
Correlation of decrease of levels of intracellular second messenger cyclic di-GMP with carbon starvation and aggregate dispersion. Aggregated growth and aggregate dispersion after glucose depletion (panel A) was monitored by OD (open circle), cellular protein (solid square), and glucose determination (solid circle). Cells collected from plankton samples were extracted, and extracts analyzed for its content in cyclic di-GMP by HPLC-MS^2^, and data plotted (panel B) as total cyclic di-GMP concentration (open circle), and cyclic di-GMP concentration relative to total cellular protein content (solid square).

Up- or down-shifts in the levels of the intracellular second messenger cyclic di-GMP have been associated with a switch between the biofilm- and planktonic lifestyle of *P. aeruginosa* [29]–[31]. Therefore, cyclic di-GMP concentrations in planktonic cultures were measured and correlated with glucose starvation and aggregate dispersal. During the growth of closely monitored 1 L cultures (Figure 7A), cellular biomass was collected at intervals and cyclic di-GMP extracted and analyzed using liquid chromatography coupled to ion-trap mass spectrometry (LC-ITMS^2^) (Figure 7B). During the growth phase, total cyclic di-GMP concentrations increased steadily concomitant with growth of the culture. However, after the culture became glucose depleted and dispersed, cyclic di-GMP levels fell to below 20% of the maximal value (cf. Figure 7AB). In contrast, when the total cyclic di-GMP content in the samples was normalized against the total cellular protein content, it appeared that the relative levels of intracellular cyclic di-GMP showed a bi-phasic decrease, with an initial decline during the growth phase, well before glucose starvation, and a second drop in concentration after glucose-starvation (Figure 7B).

### Release of active bacteriophage into the culture fluid during growth and after starvation

**Figure 8:**
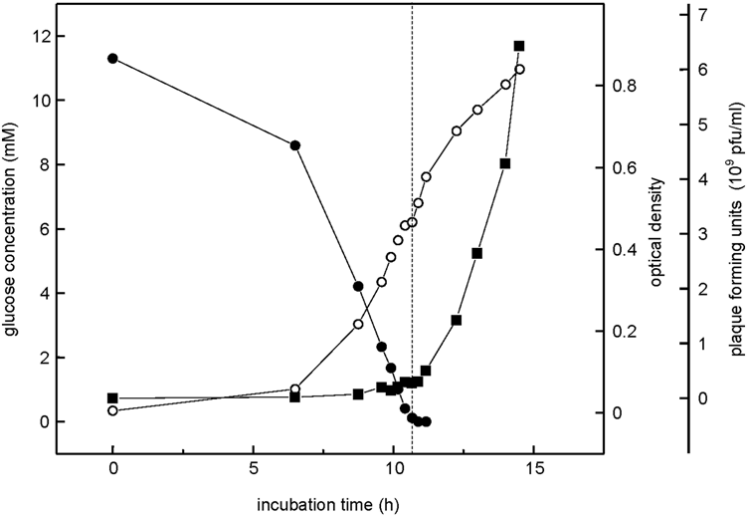
Correlation of increased release of active bacteriophage with carbon starvation. Aggregates and aggregate dispersion after glucose depletion (dashed line) was monitored by OD (open circle) and glucose determination (solid circle), and the release of active bacteriophage into the culture supernatant (square) determined as plaque formation units (pfu) after drop-plating serial dilutions of cell-free culture supernatant onto soft-agar plates with *P. aeruginosa* PAO1 ΔPf4 lawn, indicative of infective phage production.

For surface attached biofilms, the release of active bacteriophage into the culture fluid has been correlated with increased cell death and increased dispersal in mature biofilms [12], [13]. To determine if bacteriophage also plays a role in aggregate dispersal, samples collected during growth of 1 L cultures (Figure 8A) were filtered (0.2 µm pore size), the filtrates serially diluted, and drop-plated onto top-layer agar [12], which contained a phage-sensitive *P. aeruginosa* mutant as the target (Pf4-mutant, [13]) to quantify plaque forming units (pfu) (Figure 8B). The pfu counts obtained from samples that were collected during the growth phase until glucose depletion (cf. Figure 8A) remained at low levels of <0.5*10^9^ pfu/mL (0–10 h incubation time) (Figure 8B). After starvation, the pfu counts increased ten fold to 5*10^9^ pfu/mL within 3 h (Figure 8B). Samples collected in late stationary phase (>24 h incubation time; data not shown) had pfu-counts of 5*10^10^ pfu/mL, indicating continued phage release well after dispersal.

## Discussion

### Planktonic biofilm formation and starvation-induced dispersal in liquid batch cultures of *P. aeruginosa*

**Figure 9:**
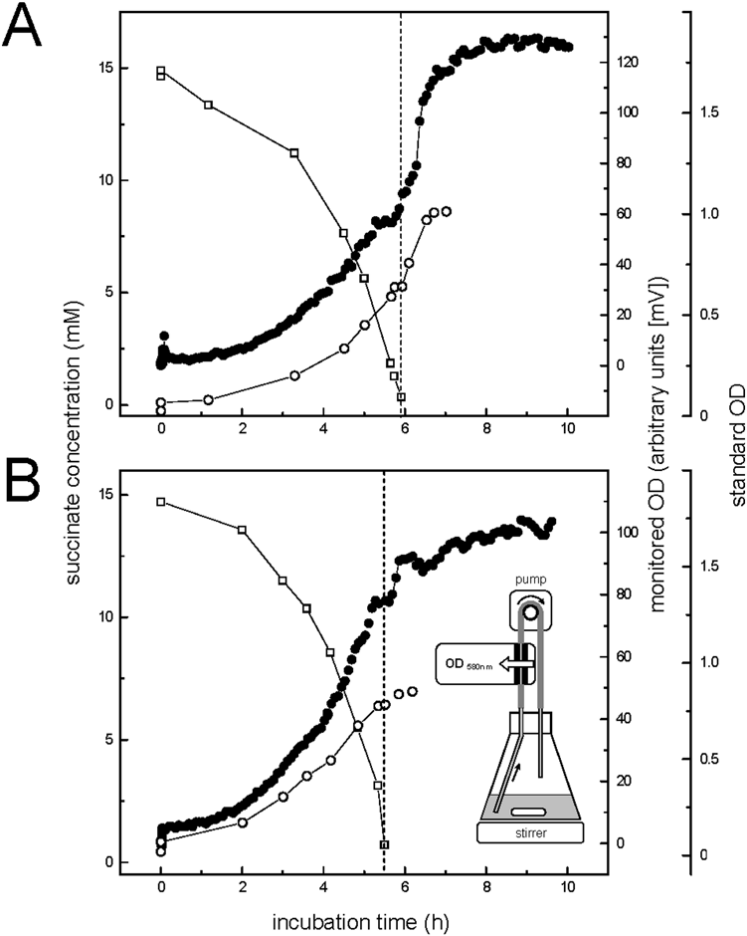
Detailed growth curves established by automated OD determination. The OD in identically incubated batch cultures of aggregating *Pseudomonas aeruginosa* PAO1 (panel A), and of non-aggregating strain *Comamonas testosteroni* KF-1 [42] for comparison (panel B), were monitored in 10-min intervals (solid circles) in a flow-through OD photometric system (see inset in panel B). At longer time intervals, growth was also followed by manual sampling and determination of standard OD (open circles) and carbon substrate concentration (open squares; succinate). Time points of starvation are indicated by dashed lines.

It is commonly referenced that the preferred mode of growth for bacteria is a high density community referred to as a biofilm [1]. The data presented here support this hypothesis and further demonstrate that this biofilm preference may even be true for planktonic cultures, where we have observed that the majority of the *P. aeruginosa* log phase population exists in aggregates with sizes between 10–400 µm diameter (cf. Figure 1). Indeed, more than 90% of the log phase population appeared to grow as aggregates under standard laboratory conditions (Figure 1BC, 6A). However, when the population experiences growth limiting conditions, e.g. carbon or nitrogen starvation, or oxygen limitation, then the aggregates rapidly disperse and release single cells. It was observed that stationary phase or starved cultures consisted primarily of single cells with less than 10% of biomass associated with aggregates (Figure 1BC, 6A). The precise time that cells became substrate limited was directly determined by monitoring glucose concentration or by switching to glucose free M9 salts. Simultaneous OD readings indicated that the OD continued to increase even after the cells had completely exhausted all available carbon, which does not fit with the fundamentals of growth physiology. This additional increase of OD, which appears as a shoulder after the onset of starvation (Figure 9), is therefore most likely due to the dispersal of suspended aggregates into dispersed, single-cells during starvation and stationary phase. The suggestion that these apparent shoulders as determined by OD (Figure 9), are the result of dispersal rather than cryptic growth is supported by our observation that there were no detectable transient carbon substrate intermediates formed during the growth of our well-aerated cultures, e.g. through the release of α-keto acids as suggestive of metabolic overflow (e.g. [32]), or for the synthesis of polyhydroxyalkanoates (PHAs) as storage polymers, to explain a “true” second growth phase in our cultures. This was further confirmed when we used total cellular protein quantification to determine growth curves (Figure 1A, 7A) when a technique was used that also collected the larger aggregates from the plankton (see Materials and Methods). Interestingly, similar increases in OD in the absence of a growth substrate have been a puzzle that was previously reported (e.g. [33]), and we presume that such dispersal phases have been termed “transition phase”, “deceleration phase”, or “reductive division”, when defined as apparent growth in the absence of a growth substrate [34], [35]. Here, we submit that these phenomena are related to substrate limitation induced dispersal of suspended aggregates and therefore are not in conflict with the principles of growth physiology.

Thus, it was demonstrated that in fact suspended biofilms occur in what have traditionally been described as planktonic, individual cell cultures of *P. aeruginosa* PAO1, that these planktonic biofilms indeed harbor the majority of the total cells of a liquid culture as opposed to individual cells, and that such planktonic biofilms have similar behaviors and responses (e.g. dispersal under limiting conditions) as do surface-attached biofilms.

### Planktonic aggregates have characteristics of surface associated biofilms

The structures of planktonic biofilms (Figure 1) were very similar to surface associated biofims of *P. aeruginosa*, containing densely packed viable cells and a fine structured extracellular polymeric matrix (EPM) consisting of extracellular DNA (eDNA) (Figure 2). For example, surface attached biofilms of *P. aeruginosa* PAO1 in continuous flow systems showed a similar fast and significant dispersal event after carbon starvation (data not shown), and is in agreement with earlier observations made with *Pseudomonas* spp. under comparable conditions [4], [5], [7], [36]. Furthermore, the dispersal event could be illustrated in biofilms that were grown as batch cultures in 12-well microtiter plates, after depletion of the single carbon source glucose (Figure 5A), or after depletion of the bulk carbon sources in complex medium (Figure 5B). When biofilms of *P. aeruginosa* were grown in batch culture on a polyester (PE) surface (see Material and Methods), a more detailed picture of the aggregation and dispersal process emerged. Growth of biofilms on the surface was initiated well before planktonic growth, which resulted in an apparent lag phase, based on optical density measurements (Figure 3B). However, this apparent lag was artificially caused by the preferential partitioning of cells onto the surface, similar to the observations reported earlier for *Pseudomonas fluorescence* cultures [36], for which nutrient limitation also induced a dispersal of surface-attached biofilms. The cells in PE-attached biofilms dispersed into the planktonic phase immediately upon carbon (Figure 3BC), nitrogen or oxygen depletion (data not shown), and the remaining biofilms on the PE surface were comprised of increased numbers of dead cells and an extracellular polymeric matrix, in particular large amounts of eDNA (Figure 4). While eDNA was detected in growing planktonic aggregates (Figure 2) and in surface attached biofilms (Figure 4), the results suggested that massive amounts of eDNA were released upon starvation concomitant to a drastic increase in dead cells (Figure 4). This increased DNA release from aggregates potentially explains the apparent slimy- and stickiness of the aggregates, and the increase of soluble DNA in the bulk fluid (data not shown), detectable after starvation. We thus suggest that the increased DNA release in the “mid-exponential growth phase” of planktonic cultures of *P. aeruginosa* PAO1 [37], was correlated with a dispersal event of an aggregated planktonic culture. Interestingly, in their study, the released DNA was confirmed to represent chromosomal DNA that was generated via lysis of a subpopulation of the bacteria, and via a mechanism dependent on important traits of the biofilm lifestyle of *P. aeruginosa*, quorum sensing (acyl homoserine lactone and *Pseudomonas* quinolone signaling) as well as on flagella and type IV pili [37]. Thus, the starvation induced dispersal event in planktonic cultures (Figure 1) was similar to dispersal of surface attached biofilms of *P. aeruginosa* PAO1 (Figure 3–5).

The observations made here suggest that starvation induces a fast release of single cells (Figure 1), with an increase in dead cells and release of eDNA (Figure 4), along with an increased release into the culture fluid of proteins, rhamnolipids, and pigments, and of superinfective bacteriophage (Figure 8). Furthermore, a decreased concentration of intracellular second messenger cyclic di-GMP could be correlated with the starvation and dispersal event (Figure 7). This is in agreement with the hypothesis that high and low levels of cyclic di-GMP serve as signal for the switch between aggregated and single-cell lifestyle, respectively [29]–[31]. Based on the understanding and the methodology provided by this study, the role of this and of other important traits of the biofilm lifestyle of *P. aeruginosa* in the observed events of planktonic biofilm formation and starvation-induced dispersal can be readily addressed in future studies, e.g. by mutational analysis.

### Implications on future studies of *P. aeruginosa*

The novel understanding that *P. aeruginosa* in standard shaken liquid culture exist as suspended biofilms during the growth phase, and actively disperses into single cells during starvation, has important implications for future studies that involve planktonic or biofilm cultures of *P. aeruginosa*. For example, when studying changes between growth and starvation, optical density measurements may not be sufficient to discriminate between these two growth phases since it is possible to observe an increase in OD, due to dispersal, even though the substrate has already been completely consumed. Moreover, in transcriptomic or proteomic studies that aim to compare surface-attached biofilms with planktonic single cells, e.g. in attempts to identify traits which are uniquely expressed during the biofilm life style of *P. aeruginosa*, but which involve samples that are taken directly from planktonic cultures (where up to 90% of the biomass may exist as aggregates), will effectively compare biofilms with biofilms. This may partly explain why to date, transcriptomic studies of *P. aeruginosa* biofilms have not identified a core set of biofilm specific genes [3]. We suggest that the transcriptomic and proteomic characterization of the two cell populations in planktonic cultures of *P. aeruginosa* PAO1, single motile cells and aggregated cells, is likely to generate a more refined picture of the sets of genes or proteins necessary to mediate biofilm formation, and the exit from biofilms e.g. after starvation. For biofilm assays, one must also carefully consider the time point of biofilm sampling relative to the time point of starvation (carbon, nitrogen or oxygen) to properly interpret the results. For example, when using 96 well microtiter plates, if one uses 24 h of biofilm growth to screen for transposon mutants involved in biofilm development, one may in fact be screening for mutants altered in dispersal since such cultures would be carbon starved and hence dispersed by that time.

### Conclusions

Based on our observations, it appears that the suspended biofilms formed in planktonic cultures of *P. aeruginosa* share many of the same features as the surface associated biofilms, including their dependencies on cyclic di-GMP, eDNA, bacteriophage, and dispersal based on carbon, nitrogen, or oxygen limitations. These data potentially explain curious observations about apparent substrate-independent increases in biomass that appear as shoulders in growth curves based on OD measurements. It is also possible that these implications hold for other biofilm forming bacteria. Therefore, it is essential to separate out suspended biofilms from true, single celled-planktonic cultures when making comparisons with biofilms. Importantly, the observations reported here can be consistently exploited in future studies of biofilm formation and dispersal processes.

## Materials and Methods

### Bacteria, culture media and growth conditions

Wild-type strain *P. aeruginosa* PAO1 and a derivative containing a chromosomal mini-Tn7 insertion of the enhanced green fluorescent protein (GFP) gene, were generously provided by Tim Tolker-Nielsen. For planktonic batch cultures (5 mL to 1 L scale), a carbon-limited mineral salts medium was used which contained carbon source (see below), 50 mM potassium phosphate buffer pH 7.2, 20 mM NH_4_Cl, 0.25 mM MgSO_4_, and trace elements solution (10 mL/L). To achieve nitrogen-limited conditions, the concentration of NH_4_Cl was reduced to 3 mM. The stock trace elements solution contained: Na_2_EDTA×2H_2_O, 500 mg/L; FeCl_2_×4H_2_O, 143 mg/L; ZnCl_2_, 5 mg/L; MnCl_2_×4H_2_O, 3 mg/L; H_3_BO_3_, 3 mg/L; CoCl_2_×6H_2_O, 20 mg/L; CuCl_2_×2H_2_O, 1 mg/L; NiCl_2_×6 H_2_O, 2 mg/L; NaMoO_4_×2H_2_O, 3 mg/L; CaCl_2_×2H_2_O, 147 mg/L. [NOTE: the concentration of CaCl_2_ (here 10 µM) was lower than typically used in M9-medium (100 µM CaCl_2_; see below), and fully supported cellular growth but limited the extent of aggregation, since aggregates >400 µm diameter interfered with LDA analysis]. Glucose, 10 mM, or 15 mM succinate or 30 mM acetate were added as carbon substrate (60 mM total C) which resulted in a final growth yield of approximately 340 mg cellular protein/L, a comparatively low biomass content which prevented the early dispersal of aggregates through oxygen-mass transfer limitation in the final growth phase (before carbon starvation; see the Results). Planktonic batch cultures (5 mL to 1 L scale) were incubated at 30°C and aerated by orbital shaking at a shaking speed (i.e apparent liquid-shear force) as indicated in the text. Cultures in the 5 mL scale were incubated in 30 mL screw-cap glass tubes (Corning) at a 45° angle in the orbital shaker. Where indicated, polyester fleece was added to increase the surface area for biofilm formation (see below). Cultures in 0.2 L or 1.0 L scale were incubated in 1 L or 5 L Erlenmeyer flasks, respectively, if not otherwise stated. All cultures were inoculated 1% with fully outgrown and dispersed pre-culture (24–48 h old).

For batch cultures in well plates, the standard M9 medium (48 mM Na_2_HPO_4_, 22 mM KH_2_PO_4_, 9 mM NaCl, 19 mM NH_4_Cl, 2 mM MgSO_4_, 100 µM CaCl_2_, pH 7.2) with 10 mM glucose as the sole carbon source, and LB medium (10 g/L tryptone, 5 g/L yeast extract, 5 g/L NaCl) were used.

### Plankton sampling from batch cultures

Wide-mouthed pipette tips (opening >3 mm) were used to avoid exclusion or shearing of cellular aggregates during the sampling. Larger aggregates sedimented quickly, and care had to be taken to ensure that aggregates were evenly suspended also during the sampling (i.e. once the shaking of the culture flasks had been paused), which was done manually by agitation to re-suspend aggregates before sampling. During growth experiments in the 1 L scale, 50 mL samples of culture fluid (after manual agitation) were withdrawn by direct pouring, and sub-samples of planktonic cells from the 50 mL sample were taken. To obtain samples of the cell-free supernatant from batch cultures, e.g. for analysis of substrate concentration and release of rhamnolipids, soluble DNA and protein, yellow/green pigments, and active bacteriophage (see below), samples (50 mL) from 1 L cultures of planktonic cells were centrifuged briefly to remove bulk cellular biomass, and filter sterilized (0.2 µm pore size).

### LDA-particle sizing of microscopical cellular aggregates in planktonic batch cultures

Laser-diffraction analysis (LDA), a method developed to investigate inorganic particle suspensions, has previously been used to monitor flocculation in activated sludge [38], and was performed using a Mastersizer/E (Malvern Instruments, Worcestershire, UK). Size-distribution scans of the planktonic cells were taken in the range of 0.5–600 µm diameter in 31 size classes. The particular settings were: stirring speed 3 (arbitrary units); laser intensity 60%, 5000 size scans, Frauenhofer count mode. The amount of planktonic suspension added to the stir-tank filled with carbon-limited mineral salts medium was adjusted to result in a LDA-obscurity of 20–30% (arbitrary units), by addition of 1–5 mL of undiluted cell suspension (using wide-mouthed pipette tips), dependent on the growth- and aggregation state. The transfer of planktonic samples and LDA-scanning were completed after 30 s of stirring, whereas liquid-shear effects on cellular aggregates in the stir-tank could be observed only when samples were re-scanned after longer intervals of stirring (i.e. after 5 min at speed 6); thus, the LDA scanning determined an accurate picture of the particle-size distribution in the parent liquid culture.

Size-distributions scans are quantified by LDA under the assumption of spherical particles, and expressed in the unit [% volume], which is the total volumetric contribution of all particles within one particular size class relative to the total volumetric contribution of all particles in all size classes. For example in this setting with single bacterial cells (average 2 µm diameter) and suspended cellular aggregates, a numerical 1∶1 mixture of (spherical) particles with the diameters 2 µm (volume 4.2 µm^3^) and e.g. 20 µm (volume 4200 µm^3^) is reported by LDA as 99.9% of the total particle volume being contributed by particles in the size class 20 µm. We considered that total (bio-)volume of particles is interchangeable with total (bio-)mass, if a tight packing of the 2-µm particles into e.g. 20-µm aggregates can be assumed, as observed here for the densely packed cellular aggregates of *P. aeruginosa* (see the Results). Thus, in this experimental series, the % bio-volume contribution of single cells (defined as size range 1–5 µm) and of cellular aggregates (size range 5–600 µm) as determined in LDA was directly related to total biomass of the plankton, as determined by e.g. total cellular protein determination.

### Sedimentation of aggregates for microscopy

Plankton samples were pipetted into a sedimentation chamber which consisted of a 2 mL Eppendorf tube that had been cut open at the base, the lid removed, seal applied to the top using silicon grease in a syringe, and glued onto a microscope slide. One mL of culture fluid (plus DNA-stain, see below) was allowed to sediment by gravity onto the surface (0.78 cm^2^) for 5 min. After sedimentation, the culture fluid was decanted (via a syringe), the sedimentation chamber removed, and a cover slip with spacers placed over the sediment-drop.

### Centrifugation-assay

Plankton samples, 1.5 mL, were transferred to a 2 mL Eppendorf tube and submitted to a short centrifugation pulse that was calibrated to yield no visible cell pellet when fully dispersed, single-cell stationary phase culture (24 h) was used, but readily visible cell pellets if aggregated plankton (10 h) was tested (here: accelerating to 2500 rpm plus 15 seconds in a Eppendorf centrifuge Model 5415). When the top-half of the sample (0.75 mL) was carefully decanted after the centrifuge-pulsing and analyzed by LDA, it appeared that all aggregates >20 µm had been removed from this fraction. Therefore, as an estimate of the relative distribution of single cells and cellular aggregates in planktonic samples, the loss of cellular biomass from the top-half of the suspension was quantified by total cellular protein determination (see below).

### Growth and quantification of surface-attached biofilms on polyester fleece

To provide an additional solid surface for biofilm formation to planktonic batch cultures (5 mL scale), polyester (PE) fleece, a three-dimensional wide-opened weave of PE fibers, was added to the culture fluid [26]. PE fleece was obtained from retail related to aquaria (ZOOBEST filter wool, Hennecken GmbH, Stolberg, Germany). For use of the material in growth experiments, PE was washed with distilled water, air dried, and the amount of PE normalized by pre-cutting (approximately 5×10×15 mm in size), and individually adjusted to a total weight of 10 mg (^+^/_−_1 mg) [the exact surface area of PE is unknown]. PE was added to 5 mL culture medium in 30 mL glass tubes prior to autoclaving. Biofilms grew firmly attached around PE fibers when tubes were incubated shaking, and PE could be removed from tubes (inclusive capillary water) to follow planktonic growth in the remaining supernatant (by OD, total protein, and substrate determination), and biofilm formation on PE (by microscopy, crystal-violet staining, and total protein determination). For microscopy, small sections of PE (and capillary water) were carefully cut out, dipped into DNA-staining solution (see below), and transferred to a microscope slide. To determine crystal-violet staining of the biofilms on PE, the whole PE fleece was collected and dipped into, i) salts medium to remove unattached cells, ii) crystal-violet staining solution (0.1% w/v,), and iii) salts medium 3 times to remove excess stain. Stained PE was blotted on a paper towel, air-dried (24 h), and the crystal violet was solubilized with 1 mL ethanol (90%, 180 rpm shaking, 12 h). Crystal violet in the ethanolic fraction was quantified at 580 nm in a spectrophotometer, and solutions diluted if A_580 nm_ was >1.5. For total protein determination of biofilm on PE, whole PE fleece was collected, blotted on a paper towel, and placed into 1 mL NaOH (0.66 M, 90°C, 6 h), and total protein determined by a Lowry-based assay (see below).

### Growth and quantification of surface-attached biofilms in well plates

Overnight cultures of *P. aeruginosa* were inoculated to 50 mL glucose/M9 medium, or LB-medium, to a final OD of 0.01. The inoculated medium was distributed to 10 different 12-well plates (IWAKI Microplate; IWAKI Glass Co) in 1.5 mL portions (triplicate), and the plates incubated on a rotary shaker at 100 rpm at 37°C. At each sampling time point, one plate was sacrificed to determine the OD of the plankton, the biofilms attached to the solid surface, and the glucose concentration. For OD measurement, 200 µL of the supernatant from each well was transferred to a 96-well plate and the OD determined in a plate reader (at 600 nm, Wallac Victor2, PerkinElmer). The remaining liquid in the wells was collected with a 5 mL sterile syringe, pooled, filter sterilized (0.22 µm pore size), and submitted to glucose determination (see below). Biofilms attached to the wells were quantified by using crystal violet (CV) staining assay. Briefly, wells were washed once with 1.5 mL of fresh M9 medium to remove loosely attached cells. After staining the biomass with 1.75 mL of CV solution (0.1% w/v), unbound CV was removed by 3 subsequent washing steps with 2 mL fresh M9 medium. Bound CV was subsequently extracted with 2 mL abs. ethanol on a rotary shaker at 150 rpm at 30°C. Finally, 200 µL were used to determine CV in a microtiter plate reader (at 580 nm, Wallac Victor^2^, PerkinElmer).

### Surface-attached biofilms in continuous flow system

Biofilms were grown with glucose/M9 medium in continuous-flow cells (dimensions 2×4×40 mm; 0.2 mL/min flow rate) and observed by microscopy for biofilm formation on the glass slide, and attached to PE fleece, which had been inserted into the flow channels (2 cm in length). Channels were inoculated with fully outgrown pre-culture (24 h old) and incubated without flow for 30 min. After start of flow and growth of biofilms, glucose starvation was applied by switching the medium flow to glucose-free M9 medium. Samples from the eluate of the flow system were collected at intervals before and after glucose starvation, and submitted to OD analysis, glucose determination, and microscopical observation.

### Staining and microscopy

For routine microscopy of plankton sediments or of aggregates attached to PE during growth experiments (see above), the biomass was stained with the membrane-permeable DNA-stain SYTO 9 (Invitrogen), using 1 µL of 3.3 mM stock solution per mL of growth medium. To stain extracellular DNA and membrane-compromised (dead) bacteria in aggregates of GFP-labeled *P. aeruginosa*, 1 µL of 1 mM stock solution of BOBO-3 stain (Invitrogen) was added to 5 mL cultures at the start of the growth experiments (incubation in the dark). Images were taken using a fluorescence microscope (Figure 1C, 2, and 3) or a confocal laser scanning microscope (Figure 4) (Olympus).

### Optical density (OD) measurement

OD (580 nm) of liquid cultures was determined irrespective of the aggregation state of the plankton when transferred by pipetting into cuvettes (1 mL, 1 cm path length) and measured immediately in a spectrophotometer (SmartSpec 3000, BioRad). OD in glass-tube cultures was determined directly in the tube (2 cm path length) in a Spectronic 20 spectrophotometer (Baush & Lomb), after PE was removed from the tube if appropriate (see above). During the course of this study, it was necessary to monitor OD in batch cultures at much closer time intervals than applicable by manual sampling, and for future studies, a flow-through OD_580 nm_ photometry system was constructed in-house (University of Konstanz workshop) and tested connected to a data-logger and computer in this study. In the used setup, culture fluid from batch cultures (100 mL scale) was continuously provided through silicon tubing to the flow-through photometer and returned to the culture flask, by a peristaltic pump at high flow speed (appr. 1 cm/sec) (see inset of Figure 9). The photometer output in [mVolts] was linearly correlated (R^2^ = 0.97) with standard OD_580 nm_ determination.

### Analysis of substrate concentration

Glucose was determined by an automated glucose-analyzer (YSI 2300 STAT PLUS, ASI Incorporated, Yellow Springs, USA) in undiluted samples of cell-free culture fluid (0.2 µm filtration). Succinate was determined by C18-reversed phase HPLC-UV.

### Total protein determination

The total cellular protein content in batch cultures was determined in a Lowry-based assay [39] using a standard curve generated from bovine serum albumin (BSA). The cellular biomass in 1 mL or 50 mL samples of culture fluid was collected by centrifugation (10 min, 16.000×*g*), resuspended in 0.6 mL or 30 mL of 0.66 M NaOH, respectively, and hydrolyzed at 90°C (6 h).

### Evaluation of rhamnolipid, soluble DNA, crude protein, and of yellow/green pigment release into the culture supernatant

Cell-free supernatant from batch cultures as collected during growth and dispersal (0.2-µm filtrates, see above) was spot-tested for increased release of the biosurfactant rhamnolipid, by strong manual shaking and observation for increased persistence of foam, indicative of surface-active compounds present in the culture fluid [40]. To quantify rhamnolipid, 10 mL of cell-free sample was extracted twice with 3 mL ethyl acetate, and pooled extracts evaporated to dryness and solubilized in 0.5 mL abs. methanol, and submitted to modified Orcinol assay [41], using rhamnose as standard. To determine soluble DNA in cell-free supernatant from batch cultures (2 mL), the nucleic-acid stain SYBR GOLD (Invitrogen) was added (0.1 mL of 500-fold diluted stock concentration) and specific fluorescence determined (excitation, 495 nm; emission, 545 nm) in a fluorescence spectrophotometer (Model LS50B, Perkin Elmer) against DNA standards in culture medium which were prepared using DNA of agarose-gel marker. To determine soluble protein in batch cultures, trichloroacetic acid (0.25 mL, 3 M) was added to cell-free supernatant (1 mL), and precipitated proteins collected by centrifugation and submitted to a Lowry-based assay (see above). The overall-release of yellow/green pigments into the supernatant fluid was monitored by eye, and quantified in cell free culture fluid as increase in absorption at 410 nm.

### Analysis of intracellular cyclic di-GMP concentration

Cyclic di-GMP was extracted from planktonic biomass after centrifugation of 50 mL samples (10 min, 12,000×*g*, 4°C), and the cell pellets were resuspended in 2 mL distilled water, incubated at 95°C for 5 min, and cooled on ice prior to addition of 3.7 mL ethanol and extended vortexing. The extraction was repeated once. Pooled extracts were evaporated to dryness in a speedvac and dissolved in 1 mL distilled water. Chloroform (1 mL) was added to the suspensions, vortexed, and separated by centrifugation (10 min, 16,000×*g*, 10°C), for collection of 0.8 mL of the aqueous phase into HPLC-vials, which were stored at −80°C until analyzed. Detection of cyclic di-GMP in cell extracts involved the Iontrap-MS/MS system THERMOFISHER LCQ Deca XP equipped with an autosampler, a quaternary pump, and an electrospray-interface. Separation of cyclic di-GMP from the bulk material contained in the extracts was achieved by reverse-phase HPLC on a Nucleosil C_18_ column (3×125 mm; particle diameter 5 µm) (Macherey-Nagel, Düren, Germany). The mobile phases were (A) water acidified with 0.1% formic acid and (B) acetonitrile with 10% water acidified with 0.1% formic acid. The gradient program was initiated at a flow rate of 200 µL/min with 100% A, and after 3 min the portion of B was linearly increased to 100% over 10 min, and maintained for 5 min. Under these conditions, cyclic di-GMP eluted at 14.2 min retention time. The column was completely eluted (waste injection) by subsequent maintenance at 100% B at a flow rate of 0.5 mL min^−1^, and re-equilibrated with 100% A for 5 min at a flow rate of 200 µL min^−1^. The injection volume was 100 µl for cell extracts. The electrospray voltage was −5 kV, the sheath gas flow 30% (arbitrary units) nitrogen gas, and the capillary was maintained at 250°C. For MS^2^ experiments, the mass width for isolation of precursor ions was 1.0 Da, and the relative collision energy was set at 30% (arbitrary units). Authentic cyclic di-GMP (Biolog, San Diego, USA) was used to optimize the HPLC separation conditions, the ESI-MS/MS^2^ - parameters, and for identification of cyclic di-GMP in cell extracts based on, i) identical elution (co-chromatography), ii) identical masses observed of molecular ions, and iii) identical MS^2^-fragmentation pattern observed of isolated precursor ions. No isotopically labeled cyclic di-GMP was available, so cyclic di-GMP in cell extracts was quantified by spiking cell extracts (stationary phase) with known concentrations of authentic cyclic di-GMP and compared against un-spiked extracts.

### Plaque forming units determination

The overlay of the bacterial lawn was prepared by mixing 500 µL of overnight cultures grown in M9 complete medium, with 5 ml of 0.8% (w/v) molten LB10 agar. The mixture was poured onto a LB10 agar plate and allowed to solidify. The plates with the filtrate samples were incubated overnight at 37°C and were observed for plaque formation. Plaque formation on the PAO1 ΔPf4 lawn indicates phage production, and plaque formation on PAO1 wild-type lawn indicates the production of superinfective form of the phage [13].
